# Effect of phospholipase A_2_ inhibitors during infection caused by *Leishmania (Leishmania) amazonensis*

**DOI:** 10.1186/s40409-018-0156-9

**Published:** 2018-08-27

**Authors:** Maria L. A. C. Bordon, Márcia D. Laurenti, Susan Pereira Ribeiro, Marcos H. Toyama, Daniela de O. Toyama, Luiz Felipe D. Passero

**Affiliations:** 1 Laboratory of Pathology of Infectious Diseases (LIM-50), Medical School, University of São Paulo (USP), Av. Dr. Arnaldo, 455, São Paulo, SP CEP 01246903 Brazil; 2 Pathology Department, Case Western Reserve University, 2103 Cornell Rd, room 5503, Cleveland, OH 44106 USA; 3 São Paulo State University (UNESP), Institute of Biosciences, São Vicente, Praça Infante Dom Henrique, s/n, 11330-900 São Vicente, SP Brazil; 4 School of Dentistry, Camilo Castelo Branco University (Unicastelo), Rua Carolina Fonseca, 584, São Paulo, SP CEP 08230-030 Brazil

**Keywords:** *Leishmania (Leishmania) amazonensis*, Macrophages, BALB/c mice, Phospholipase A_2_, Phospholipase A_2_ inhibitors

## Abstract

**Background:**

Lipid metabolites play an important role in parasite differentiation and virulence. Studies have revealed that *Leishmania* sp. uses prostaglandins to evade innate barriers, thus enabling the parasites to survive inside immune cells. Despite the role of the enzyme Phospholipase A_2_ (PLA_2_) in prostaglandins production, few studies have investigated the role of parasite PLA_2_ during the interaction between *L. (L.) amazonensis* and the host (in vitro and in vivo) immune cells.

**Methods:**

In the present work, the leishmanicidal effect of PLA_2_ inhibitors, methyl arachidonyl fluorophosphonate (MAFP), bromoenol lactone (BEL) and aristolochic acid (AA) were investigated in vitro (promastigote and intracellular amastigote forms of *L. (L.) amazonensis*) and during in vivo infection using BALB/c mice.

**Results:**

The aforementioned inhibitors were deleterious to promastigote and amastigote forms of the *L. (L.) amazonensis* and were non-toxic to peritoneal macrophages from BALB/c mice. *L. (L.) amazonensis*-infected BALB/c mice treated with the inhibitor BEL presented decreased lesion size and skin parasitism; however, BEL treatment induced hepatotoxicity in BALB/c mice.

**Conclusions:**

Results presented herein suggested that PLA_2_ inhibitors altered *L. (L.) amazonensis* viability. In spite of liver toxicity, treatment with BEL was the most selective compound in vitro, as well in vivo, resulting in lower skin parasitism in the infected mice. These findings corroborate the role of PLA_2_ in parasite virulence and maintenance in vertebrate hosts, and suggest that molecules structurally related to BEL should be considered when planning compounds against *Leishmania* sp.

## Background

The *Leishmania* parasite and its first steps of interactions with phagocytic cells have been extensively studied, mainly because the type of interaction and molecules involved determine the fate of *Leishmania*, which may be associated with death by molecules and/or cells from the host; or *Leishmania* parasites can enter in the main host’s cells, the macrophages, determining disease [1]. The initial interaction between macrophages with the *Leishmania* sp. occurs through the complement receptor (CR), mannose-fucose, fibronectin, and Fcγ macrophage receptors.

Following inoculation of *Leishmania* promastigotes into the dermis of the mammalian host, a parasite metalloproteinase of 63 kDa (gp63) is able to cleave the C3b factor of the complement system into an inactive form (iC3b), which is able to bind to leishmanial lipophosphoglycan (LPG) and even to gp63. These opsonized *Leishmania* promastigotes bind to CR1 and CR3 macrophage receptors thereby commencing phagocytosis. This main type of phagocytosis seems to impact the course of infection, since the inhibition of respiratory burst and the Th1-driven immune response create favorable conditions for *Leishmania* survival. Conversely, interactions between *Leishmania* and fibronectin receptors will trigger an inflammatory response associated with parasite death [2, 3].

Leishmanial molecules are also critical in the modulation of macrophages’ intracellular environments. LPG is one of the main glycoconjugate of *Leishmania* promastigotes, and is involved in the protection of the parasite not only from the acidic parasitophorous vacuoles, but also from inhibition of phagosome maturation and modulation of cytokine production. The gp63 metalloproteinase has been credited as being a potent inhibitor of the protein kinase C pathways, which, if functioning properly, are responsible for cell proliferation, differentiation, apoptosis, and reactive oxygen and nitrogen species production; this context suggests that gp63 (in addition to LPG) has a deep impact on the modulation of leishmanicidal activity and in the establishment of leishmanial infection in macrophages [4–6]. These types of studies are extremely important for expanding the current knowledge on the physiopathology of leishmaniasis.

Although LPG and gp63 antigens have been identified as vital for parasite survival, other parasitic components are also important during the process of phagocytosis, as well as in the intracellular survival of *Leishmania* parasites. In this regard, it was demonstrated that the supernatant of *L. (L.) amazonensis* presented phospholipase A_2_ (PLA_2_) activity, and when an additional source of PLA_2_ was added in culture, the pathway of eicosanoid production was stimulated and prostaglandin E_2_ (PGE_2_) was produced at high levels; this was associated with increased numbers of intracellular amastigotes [7]. Moreover, in vivo studies demonstrated that PLA_2_-stimulated *L. (L.) amazonensis* induced tissue injuries when compared with the control parasite [7]. This suggests the involvement of PLA_2_ in the pathway of prostaglandin production, and that this pathway can be considered an additional mechanism by which *L. (L.) amazonensis* parasites infect, modulate inflammation and persist in the host.

Overall PGE_2_, a major byproduct of the metabolism of arachidonic acid, has been linked to pathology in leishmaniasis. Farrel and Kirkpatrick [8] were among the first to suggest the participation of this lipid mediator in leishmaniasis, since *L. major*-infected splenocytes from BALB/c mice produced elevated amounts of PGE_2_ and were unable to proliferate under specific stimuli. This is in contrast to splenocytes isolated from animals treated with indomethacin, a selective inhibitor of the enzyme cyclooxygenase (COX), which did not produce PGE_2_ and were capable of proliferating. Similarly, the human lineage of U937 macrophages presented a time-dependent elevation in the production of PGE_2_ following infection with *L. donovani* [9], and decreased after the addition of COX_2_ inhibitors. On the other hand, other secreted PLA_2_ enzymes were able to eliminate promastigote forms of *L. (L.) infantum*, *L. (L.) amazonensis* [10–12]*.*

In order to evaluate the importance of PGE_2_ during experimental leishmaniasis, *L. (L.) mexicana*-infected BALB/c mice were treated with indomethacin. It was observed that treated animals partially controlled the size of lesions and skin parasitism parasite load the skin, a finding that was associated with reduced levels of interleukin (IL)-4, IL-10, and PGE_2_ in the supernatants of splenocytes [13]. These works clearly demonstrated how prostaglandin production can modulate not only the immunological response but also the outcome of parasite infection, suggesting that certain inhibitors of this complex pathway can serve as useful tools for controlling parasitism – such as the inhibitors of PLA_2_, which is a key enzyme responsible for triggering prostaglandin production. Thus, the main purpose of this work is to investigate the efficacy of inhibitors of cytosolic and secreted PLA_2_, such as bromoenol lactone (BEL), methyl arachidonyl fluorophosphonate (MAFP) and aristolochic acid (AA) during infection (in vitro and in vivo) with *L. (L.) amazonensis*.

## Methods

### Parasites

The *L. (L.) amazonensis* parasite (MHOM/BR/73/M2269) was kindly provided by Prof. Dr. Fernando T. Silveira from the Leishmaniasis Laboratory Prof. Dr. Ralph Laison Cryobank, Department of Parasitology, Evandro Chagas Institute, Ministry of Health, Belém, Pará, Brazil. Parasite phenotyping was identified by monoclonal antibodies and isoenzyme electrophoretic profiles (at the Leishmaniasis Laboratory of the Evandro Chagas Institute - Belém, Pará, Brazil). Parasites were grown in RPMI 1640 medium (Roswell Park Memorial Institute - Gibco®; Thermo Fisher Scientific, Waltham, MA, USA), supplemented with 10% heat-inactivated fetal bovine serum, 10 μg/mL of gentamicin, and 1000 U/mL of penicillin (R10) at 25 °C. Promastigote forms in the stationary phase were used.

### PLA_2_ inhibitors

Aristolochic acid (AA) inhibits secretory PLA_2_, such as human synovial fluid PLA_2_ and PLA_2_ purified snake and scorpion venoms. Bromoenol lactone (BEL) is an irreversible inhibitor of calcium-independent PLA_2_ able to inhibit the release of arachidonate from different cell lines. Methyl arachidonyl fluorophosphonate (MAFP) is a selective and irreversible inhibitor of cytosolic PLA_2_ and calcium-independent PLA_2_.

### Determination of leishmanicidal potential of PLA_2_ inhibitors

Promastigote forms of *L. (L.) amazonensis* (2 × 10^6^ promastigotes/well) were incubated in 96-well culture plates in R10 medium with bromoenol lactone (BEL) (0.7–60 μM), methyl arachidonyl fluorophosphonate (MAFP) (4.7–300 μM) or Aristolochic acid (AA) (9.4–600 μM). Miltefosine was used as standard drug (1.9–245 μM). A negative control group cultivated in medium and dimethyl sulfoxide (DMSO) was used as a vehicle solution (never exceeding 1% *v*/v). The parasites were incubated for 24 h at 25 °C. Plates were washed 3 times with 200 μL of sodium chloride 0.9% (*w*/*v*) and centrifuged at 3000 rpm for 10 min at 4 °C; MTT (3-[4,5-dimethylthiazol-2-yl]-2,5-diphenyltetrazolium bromide) (5.0 mg/mL) was added for 4 h. Subsequently, 50uL of sodium dodecyl sulfate (SDS -10%) was added to each well. The plates were incubated for 18 h and read in an enzyme-linked immunosorbent assay (ELISA) reader at 595 nm. In order to access the leishmanicidal potential of PLA_2_ inhibitors, 50% effective concentrations (EC_50_) were estimated using the software Graph Pad Prism 5.0 (GraphPad Software Inc., La Jolla, CA, USA). The EC_50_ is the concentration of the inhibitors where 50% of leishmanicidal effect is observed after the specified exposure time.

### Peritoneal macrophage culture and cytotoxicity assay

Approximately 2 × 10^5^ peritoneal macrophages from BALB/c mice were cultured in R10 medium with BEL (0.7–60 μM), MAFP (4.7–300 μM), AA (9.4–600 μM) or miltefosine (1.9–245 μM). As negative control, macrophages were cultivated in medium and DMSO (not exceeding 1% *v*/v). After 24 h, cell viability was analyzed by the MTT method. After evaluation of the surviving curves, non-toxic concentrations were recorded and used in further experiments. In addition, the 50% cytotoxic concentration (CC_50_) was estimated using the software GraphPad Prism 5.0. The CC_50_ is defined as the concentration of the inhibitors where 50% of the host cells were unviable after the specified exposure time.

### Effect of PLA_2_ inhibitors during the interaction between the parasite and host macrophages

Promastigote forms of *L. amazonensis* in stationary phase were adjusted to a concentration of 2 × 10^6^ promastigotes/mL and were added to macrophage cultures (1:10 macrophage-to-parasite ratio). The co-cultures were maintained in a humidified incubator at 5% CO_2_ at 35 °C. The inhibitors AA (25.0; 50.0; 100.0 μM), BEL (1.0; 2.0; 4.0 μM) and MAFP (5.0; 10.0; 20.0 μM) were added to the infected cells. As standard treatment, miltefosine’s EC_50_ was used [14]. After 24 h of incubation, infection indexes were estimated [15] and the concentrations able to decrease the infection index to 50% were estimated via the software GraphPad Prism 5.0.

### Efficacy of PLA_2_ inhibitors during experimental cutaneous leishmaniasis

Thirty male BALB/c mice were subcutaneously infected in the right hind footpad with 10^6^ promastigote forms of *L. (L.) amazonensis*, while five BALB/c mice received sodium chloride 0.9% (*w*/*v*) via the same route (healthy group). Five weeks after infection, *L. (L.) amazonensis-*infected BALB/c mice were divided into six groups containing 5 animals each: groups 1 and 2 were injected with 10.0 nM (0.012 μg/kg) and 30.0 nM (0.036 μg/kg) of BEL, respectively. The concentration of BEL was chosen based on previous reports of animal treatment [16]; groups 3 and 4 were injected with 0.7 mM (0.015 μg/kg) and 1.4 mM (0.030 μg/kg) of MAFP, respectively. All these groups were treated intraperitoneally. Group 5 received Glucantime (50 mg/kg) intralesionally. Group 6 (infected only) was injected with PBS solution. Group 7 (non-infected, non-treated animals) received only the vehicle solution (PBS control by intraperitoneal or subcutaneous routes).

Animals treated intraperitoneally were injected with 50 μL of PLA_2_ inhibitors or PBS solution; while those treated intralesionally received 20 μL of Glucantime or PBS solution. Animals were injected with PLA_2_ inhibitors, Glucantime or vehicle solution a total of 15 times, once a day, at 24-h intervals. The animals’ physical conditions were monitored once a week. Glucantime was injected intralesionally, based on a previous work [17], and this administration route was effective at eliminating tissue amastigotes. The inhibitors BEL and MAFP were injected intraperitoneally to improve their distribution in the animal body. A group of non-infected animals were treated with BEL, MAFP or Glucantime to analyze histological alterations. One week after the last injection, the animals were anaesthetized with thiopental and sacrificed by cardiac puncture. There were no deaths prior to the endpoint, and all animals were euthanized to analyze the parasitism in the skin. Animals were not treated with AA because reports in the literature had reported its high toxicity for animals [18].

### Clinical course of lesion development and determination of parasite burden on the skin and in lymph nodes

The development of lesions in the infected and treated groups was measured weekly following infection for 8 weeks. The size of the lesion was determined based on the difference between infected and uninfected footpads. The parasite loads in the skin and in lymph nodes were determined using the quantitative limiting-dilution assay, as described in [19]. Briefly, fragments from the infected footpads and lymph nodes of different groups were aseptically excised and homogenized in Schneider’s medium. The skin and lymph node suspensions were subjected to 12 serial dilutions with four replicate wells. The number of viable parasites was determined based on the highest dilution in which the promastigotes could be grown after 10 days of incubation at 25 °C. Biopsies of the heart, lung, spleen, liver and kidney were collected and fixed in buffered 5% formalin for analysis of histopathological alterations in treated animals. These organs were collected because they are highly vascularized, which enables drugs in the bloodstream to provoke major changes and impact their physiology.

### Statistical analysis

The results were expressed as the mean ± standard deviation of three independent experiments, and the nonparametric Mann-Whitney *U* test was used to compare the results between groups. Differences were considered statistically significant at a 5% significance level (*P* < 0.05). The software GraphPad Prism 5 was employed to analyze the results.

## Results

### Leishmanicidal and cytotoxic activities of PLA_2_ inhibitors

BEL was the most active PLA_2_ inhibitor, killing 50% of promastigote forms of *L. amazonensis* with 15.1 ± 3.7 μM; MAFP presented intermediate activity (50.5 ± 7.8 μM), followed by AA, eliminating promastigote forms with EC_50_ of 450.1 ± 45.6 μM; miltefosine eliminated promastigote forms with an EC_50_ of 12.6 ± 2.1 μM (Table 1). PLA_2_ inhibitors did not induce cytotoxicity to peritoneal macrophages in the tested range (Table 1). The most selective molecule was MAFP, followed by miltefosine and BEL. The lowest selectivity index was presented by AA (Table 1).

**Table 1:** Activity of PLA_2_ inhibitors against promastigote, amastigote and peritoneal macrophages from BALB/c mice

Compounds	EC_50_ (μΜ)^a^	CC_50_ (μM)	SI^a^	EC_50_ (μM)^b^	SI^b^
AA	450.1 ± 45.6	> 600	> 1.3	76.9 ± 5.7	> 7.8
BEL	15.1 ± 3.7	> 60	> 3.9	2.6 ± 0.8	> 23.1
MAFP	50.5 ± 7.8	> 300	> 5.9	17.6 ± 7.9	> 17.0
Miltefosine	12.6 ± 2.1	60.8 ± 3.1	4.8	21.6 ± 2.2	2.8

The efficacies of compounds were analyzed through effective concentration 50 (EC_50_), and the selective indexes (SI) were estimated

^a^ promastigote; ^b^ amastigote

It was observed that BEL was the most active against amastigote forms and decreased the infection index by 50% at 2.6 ± 0.8 μM, followed by MAFP (17.6 ± 7.9 μM), miltefosine (21.6 ± 2.2 μM) and AA (76.9 ± 5.7 μM), as indicated in Table 1. Furthermore, it was observed that BEL was the most selective molecule, followed by MAFP, AA and finally miltefosine (Table 1).

### Effect of PLA_2_ inhibitors during experimental cutaneous leishmaniasis

**Fig. 1:**
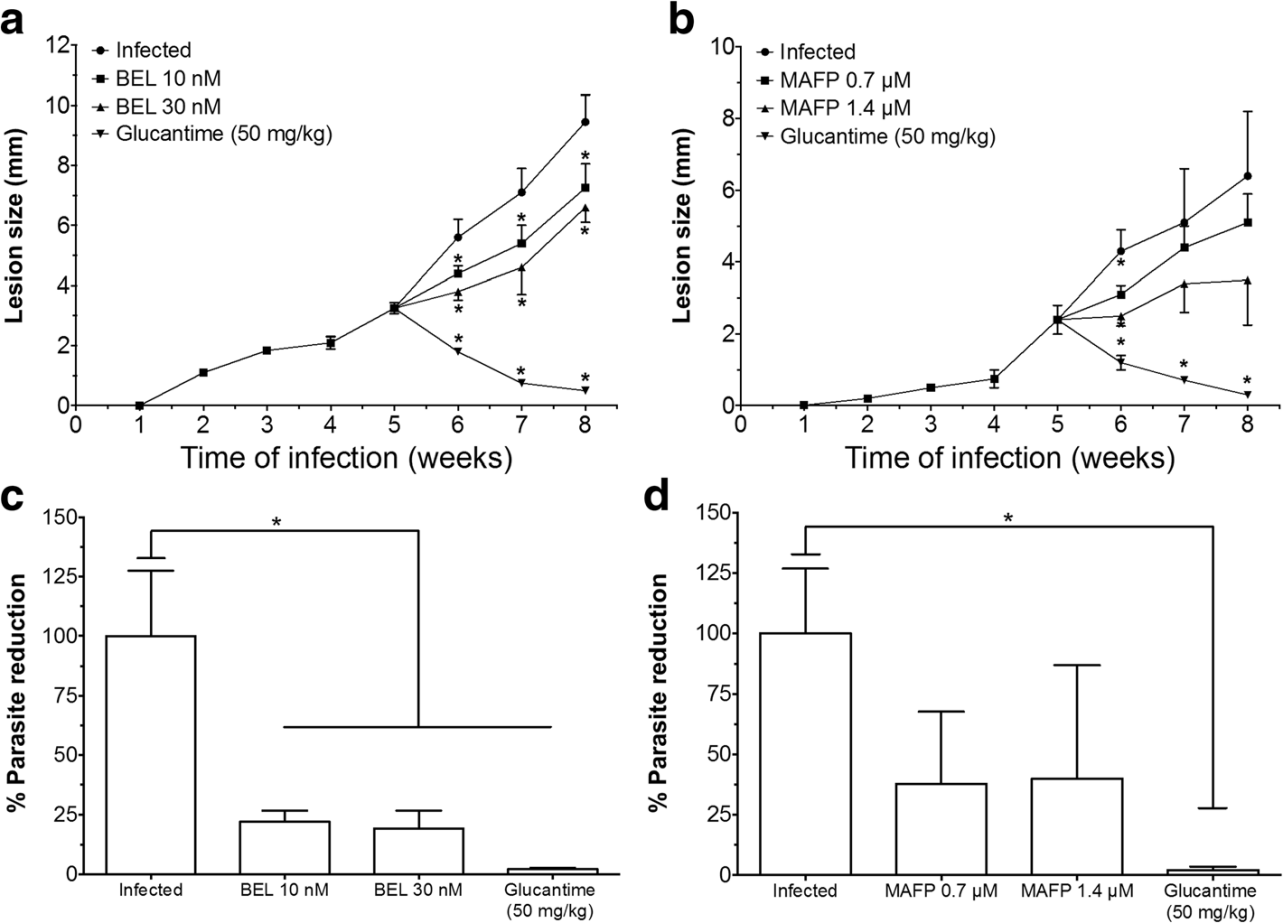
BALB/c mice were infected in the right hind footpad with promastigote forms of *L. amazonensis* in stationary phase of growth. Five weeks after infection, the treatments were started and the lesion sizes of BEL- and MAFP-treated animals were recorded (**a** and **b**, respectively), and the skin parasitism analyzed in the animals treated with BEL (**c**) and MAFP (**d**). *P<0.05 indicates significant differences when comparing the treated groups versus infected control group

Infected BALB/c mice treated with 10 nM or 30 nM of the inhibitor BEL (Fig. 1a) presented smaller lesion sizes at weeks 6, 7, and 8 post-infection (*P* < 0.05), when compared with the infected control. In contrast, infected animals treated with 0.7 or 1.4 μM of MAFP (Fig. 1b) did not show significant alterations during the course of infection. Animals treated intralesionally with Glucantime presented small lesion size from 6 weeks post-infection until the end of the experiment, at the 8th week (Fig. 1a and b).

In relation to skin parasitism, animals treated with 10 nM or 30 nM of BEL had fewer parasites on the skin when compared with the infected control (Fig. 1c). Infected animals treated with MAFP did not change the parasite load in the skin (Figs. 1d). Glucantime-treated animals (50 mg/kg) presented decreased parasite load when compared with the infected control group (Figs. 1c and d). Animals treated with BEL or MAPF presented lymph node parasitism similar to that in the infected control group (data not shown). Parasites were not detected in lymph nodes of Glucantime-treated animals (data not shown).

### Histopathological analysis

**Fig. 2:**
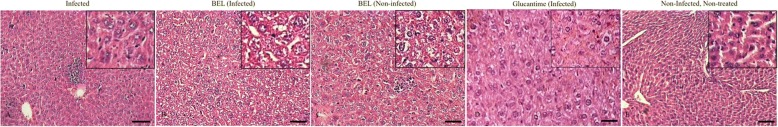
Histopathology analysis of the liver section. Liver of infected control (**a**) showed portal inflammation; the inset shows preserved hepatocytes and Kupfer cells. Infected (**b**) and non-infected (**c**) animals treated with 30 nM of BEL showed degeneration of hepatocytes (details in the insets). Liver of infected animals treated with Glucantime did not show signs of histological changes (**d**). Histological section of the liver from healthy animals (**e**)

Histological sections of the heart, liver, kidney, and spleen were analyzed to verify toxic effects of PLA_2_ inhibitors on BALB/c mice. Infected control animals did not present significant histolopathological changes in the heart, kidney or lungs; however, in the spleen signals of germinal center activation were verified (data not shown). In the liver, some focal areas of inflammation were observed (black arrow in Fig. 2a).

Infected animals treated with MAFP did not show significant changes in analyzed organs compared with the infected control (data not shown). In comparison with infected controls, the BEL-treated group did not present signs of alterations in the heart, spleen or lungs (data not shown); however, the liver presented extensive areas of hepatocellular necrosis, as illustrated in the insets of one respective image from the group treated with 30 nM of BEL (Fig. 2b). A similar histological pattern was verified in non-infected animals treated with 30 nM of BEL (Fig. 2c). Infected and non-infected animals treated intralesionally with Glucantime did not show changes in histological sections of analyzed organs; for comparisons, a histological liver section from an infected animal treated with 50 mg/kg of Glucantime is presented in Fig. 2d. Healthy animals did not present alterations in analyzed organs (data not shown); for comparisons a histological liver section is presented in Fig. 2e.

## Discussion

In spite of classical mechanisms of innate immunity during the relation between parasite and host cells, other components of the inflammatory process may take place during *Leishmania* infection. Recently, it was demonstrated that *L. (L.) amazonensis*-infected macrophages treated with PLA_2_ presented more intracellular amastigotes when compared with the control group. Furthermore, an association between high intracellular parasitism and PGE_2_ production by infected macrophages was demonstrated [7]; PGE_2_ is a lipidic mediator that downregulates the respiratory burst in infected macrophages [20]. Other studies have also demonstrated a pathogenic role of the prostaglandin pathway in leishmaniasis [21]; however, to the best of our knowledge, few studies have focused on the initial enzyme responsible for triggering prostaglandin production, the PLA_2_ enzyme, during experimental leishmaniasis.

Thus, in the present study, it was demonstrated that the viability of *L. (L.) amazonensis* promastigotes was altered when the PLA_2_ inhibitors BEL and MAFP were added in culture. In trypanosomatids, PLA_2_ plays vital roles in host cell invasion, Ca^+ 2^ influx, and during lipid turnover [22, 23]. Therefore, these inhibitors should affect the physiology of *L. (L.) amazonensis*, thus impacting their survival in culture, as evidenced in Table 1. In addition, *L. (L.) amazonensis* promastigotes were more sensitive to the inhibitor BEL in comparison with other treatments, and this molecule is a specific inhibitor of Ca^2+^-independent PLA_2_, since 15.1 μM was able to eliminate 50% of the parasite population; followed by MAFP, an inhibitor of calcium-dependent PLA_2_, that presented an EC_50_ of 50.5 μM. These data suggest that Ca^2+^-independent PLA_2_ may hold vital biological significance for the survival of parasites, such that molecules able to target these enzymes can be considered valuable prototype drugs. Moreover, the activities of these inhibitors on the parasites seems to be specific, since macrophages incubated with these same inhibitors, at the same range of concentrations, did not show significant alterations in their viability, suggesting that the inhibitors are more selective to the parasite than to the host cells.

In order to verify whether PLA_2_ inhibitors can impact the survival of intracellular amastigotes, *L. (L.) amazonensis-*infected macrophages were treated with BEL, MAFP and AA. In this case, both BEL and MAFP inhibitors were able to decrease the intracellular parasitism, suggesting that different families of PLA_2_ enzymes not only may play some role during the infection, but also might be important for the intracellular survival of amastigote forms. Possibly PLA_2_s (from host and parasites) may favor the production of PGE_2_, which is suppressive for macrophages [24, 25], as demonstrated in different studies [7, 9, 26, 27]. In the other hand, AA was shown to be the least active of all inhibitors assayed, despite presenting an intermediate selectivity index. Other PLA_2_ inhibitors – such as quinacrine, 4-bromophenacyl bromide and phentermine – were used during the interaction of *Trypanosoma cruzi* and macrophages. In this case it was observed that the inhibitors suppressed parasite surface binding on the host cell and internalization [28], suggesting that PLA_2_ presents a role during intracellular infections, and thus can be used as a molecular target for designing new leishmanicidal molecules.

Given that the inhibitors BEL and MAFP were able to decrease the intracellular parasitism into host macrophages, in vivo experiments were carried out to verify whether PLA_2_ could be considered a molecular target to characterize new prototype drugs. In this aspect, it was verified that BALB/c mice infected with *L. (L.) amazonensis* and treated with 10 nM or 30 nM of BEL exhibited decreased lesion size at 6, 7, and 8 weeks post-infection when compared with the infected control, an effect associated with reduced parasitism on the skin, but not in the lymph nodes (data not shown), suggesting that in vivo PLA_2_ may be responsible, at least in part, for the induction of pathology in a murine model of cutaneous leishmaniasis. On the other hand, animals treated with MAFP did not show significant alterations in the course of infection. The inhibitor AA was not assayed in vivo due to its toxicity for animals [14].

In the cellular environment, PLA_2_ enzymes catalyze the hydrolysis of phospholipid sn-2 ester bond from cell membranes. This reaction is the primary pathway that allows the release of arachidonic acid. Following this biochemical step, the enzyme cyclooxygenase converts arachidonic acid into different classes of prostaglandins, according to the available enzymes associated with eicosanoid production. In leishmaniasis some studies demonstrated that this pathway is correlated with disease worsening; on the other hand, the use of inhibitors or commercial available drugs able to interfere with enzymes pertaining to this pathway can suppress the production of prostaglandins, thus improving the lesions. In this respect, Pérez-Santos and collaborators [13] showed that the treatment of *L. (L.) mexicana*-infected BALB/c mice with indomethacin, a selective inhibitor of COX enzymes, was able to restrain skin and lymph node parasitism; moreover, treated animals presented increased amounts of Th1 interleukins [13, 29]. Therefore, the blockage of enzymes belonging to this pathway can improve the outcome of infection. Given that PLA_2_ is the first step in triggering eicosanoid production, it should be considered an interesting molecular target for designing new therapeutic molecules.

In spite of these positive findings, the inhibitor BEL was toxic to the liver of infected BALB/c mice as well as their non-infected counterparts, as demonstrated in histological sections, where degenerated hepatocytes (inset) and necrotic areas were identified. A previous work demonstrated that although BEL was not toxic to peritoneal macrophages, but the constant inhibition of PLA_2_ in the neurons resulted in a long-term loss of neuronal viability, suggesting that in some organs, (such as the liver), preserving the activity of PLA_2_s is essential for maintaining host cell viability and homeostasis [30]. This study also opens future perspectives to use BEL inhibitor as an intralesional treatment, which in turn could reduce liver toxicity.

Despite the toxicity presented by BEL, the relevance of PLA_2_ during the infection was also demonstrated. Furthermore, although BALB/c mice did not clear the *L. (L.) amazonensis* infection following the BEL treatment, these results suggested that PLA_2_ might represent a potential target for inhibiting *Leishmania* infection. However, the main challenge is to find or design effective and non-toxic PLA_2_ inhibitors that are able to block the entry or survival of the leishmania parasites in host cells.

## Conclusions

Taken together, our results demonstrated that promastigote and intracellular amastigotes (in vitro and in vivo) of *L. (L.) amazonensis* are more sensitive to BEL inhibitor compared with other inhibitors, and that molecules structurally correlated with BEL might serve as an interesting alternative for designing new prototypes directed against *Leishmania* parasites.

## Abbreviations

%: Percentage
°C: Celsius
AA: Aristolochic acid
BEL: Bromoenol lactone
COX: Cyclooxygenase
CR: Complement receptor
CR1 and CR3: Complement receptors 1 or 3, respectively
ELISA: Enzyme-linked immunosorbent assay
gp63: Metalloproteinase of 63 kDa
iC3b: Inactive form of C3b fragment
IL: Interleukin
kDa: Kilo Dalton
kg: Kilogram
*L.*: *Leishmania*
LPG: Lipophosphoglycan
MAFP: Methyl arachidonyl fluorophosphonate
MTT: 3-[4,5-dimethylthiazol-2-yl]-2,5-diphenyltetrazolium bromide
nM: Nanomolar
PGE_2_: Prostaglandin E_2_
PLA_2_: Phospholipase A_2_
R10: RPMI medium
Th1: T helper 1
μg: Microgram
μL: Microliter
μM: Micromolar

## References

[1] de Morais CGV, Castro Lima AK, Terra R, dos Santos RF, Da-Silva SAG, PML D (2015). The dialogue of the host-parasite relationship: *Leishmania* spp and *Trypanosoma cruzi* infection. Biomed Res Int.

[2] Arango Duque G, Descoteaux A (2015). *Leishmania* survival in the macrophage: where the ends justify the means. Curr Opin Microbiol.

[3] Podinovskaia M, Descoteaux A (2015). *Leishmania* and the macrophage: a multifaceted interaction. Future Microbiol.

[4] Olivier M, Gregory DJ, Forget G (2005). Subversion mechanisms by which *Leishmania* parasites can escape the host immune response: a signaling point of view. Clin Microbiol Rev.

[5] Forestier CL, Gao Q, Boons GJ (2014). *Leishmania* lipophosphoglycan: how to establish structure-activity relationships for this highly complex and multifunctional glycoconjugate?. Front Cell Infect Microbiol.

[6] Passero LFD, Assis RR, da Silva TNF, Nogueira PM, Macedo DH, Pessoa NL (2015). Differential modulation of macrophage response elicited by glycoinositolphospholipids and lipophosphoglycan from *Leishmania* (*Viannia*) *shawi*. Parasitol Int.

[7] Passero LFD, Laurenti MD, Tomokane TY, Corbett CEP, Toyama MH (2008). The effect of phospholipase A2 from *Crotalus durissus collilineatus* on *Leishmania (Leishmania) amazonensis* infection. Parasitol Res.

[8] Farrell JP, Kirkpatrick CE (1987). Experimental cutaneous leishmaniasis. II. A possible role for prostaglandins in exacerbation of disease in *Leishmania* major-infected BALB/c mice. J Immunol.

[9] Matte C, Maion G, Mourad W, Olivier M (2001). *Leishmania donovani*-induced macrophages cyclooxygenase-2 and prostaglandin E2 synthesis. Parasite Immunol.

[10] Barros GAC, Pereira AV, Barros LC, Lourenço A, Calvi SA, Santos LD (2015). *In vitro* activity of phospholipase A2 and of peptides from *Crotalus durissus terrificus* venom against amastigote and promastigote forms of *Leishmania (L.) infantum chagasi*. J Venom Anim Toxins incl Trop Dis.

[11] Terra ALC, Moreira-Dill LS, Simões-Silva R, Monteiro JRN, Cavalcante WLG, Gallacci M (2015). Biological characterization of the Amazon coral *Micrurus spixii* snake venom: isolation of a new neurotoxic phospholipase A2. Toxicon.

[12] Pereira AV, de Barros G, Pinto EG, Tempone AG, de Oliveira Orsi R, dos Santos LD (2016). Melittin induces *in vitro* death of *Leishmania* (*Leishmania*) *infantum* by triggering the cellular innate immune response. J Venom Anim Toxins incl Trop Dis.

[13] Pérez-Santos JL, Talamás-Rohana P (2001). *In vitro* indomethacin administration upregulates interleukin-12 production and polarizes the immune response towards a Th1 type in susceptible BALB/c mice infected with *Leishmania mexicana*. Parasite Immunol.

[14] Bezerra-Souza A, Yamamoto ES, Laurenti MD, Ribeiro SP, Passero LFD (2016). The antifungal compound butenafine eliminates promastigote and amastigote forms of *Leishmania* (*Leishmania*) *amazonensis* and *Leishmania* (*Viannia*) *braziliensis*. Parasitol Int.

[15] Passero LFD, Sacomori JV, Tomokane TY, Corbett CEP, da Silveira FT, Laurenti MD (2009). *Ex vivo* and *in vivo* biological behavior of *Leishmania* (*Viannia*) *shawi*. Parasitol Res.

[16] Yeo JF, Ong WY, Ling SF, Farooqui AA (2004). Intracerebroventricular injection of phospholipases A_2_ inhibitors modulates allodynia after facial carrageenan injection in mice. Pain.

[17] Yamamoto ES, Campos BLS, Jesus JA, Laurenti MD, Ribeiro SP, Kallás EG (2015). The effect of ursolic acid on *Leishmania* (*Leishmania*) *amazonensis* is related to programed cell death and presents therapeutic potential in experimental cutaneous leishmaniasis. PLoS One.

[18] Shi M, Ma L, Zhou L, Fu P. Renal protective effects of 17β-estradiol on mice with acute aristolochic acid nephropathy. Molecules. 2016;21(10):1391. 10.3390/molecules21101391 PMC6274086 27763560

[19] Campos BLS, Silva TN, Ribeiro SP, Carvalho KIL, Kallás EG, Laurenti MD (2015). Analysis of iron superoxide dismutase-encoding DNA vaccine on the evolution of the *Leishmania amazonensis* experimental infection. Parasite Immunol.

[20] Serezani CH, Chung J, Ballinger MN, Moore BB, Aronoff DM, Peters-Golden M (2007). Prostaglandin E_2_ suppresses bacterial killing in alveolar macrophages by inhibiting NADPH oxidase. Am J Respir Cell Mol Biol.

[21] Arcanjo AF, LaRocque-de-Freitas IF, Rocha JDB, Zamith D, Costa-da-Silva AC, Nunes MP (2015). The PGE2/IL-10 axis determines susceptibility of B-1 cell-derived phagocytes (B-1CDP) to *Leishmania major* infection. PLoS One.

[22] Opperdoes FR, van Roy J (1982). The phospholipases of *Trypanosoma brucei* bloodstream forms and cultured procyclics. Mol Biochem Parasitol.

[23] Belaunzarán ML, Lammel EM, de Isola ELD (2011). Phospholipases a in trypanosomatids. Enzyme Res.

[24] Tsuchida K, Ibuki T, Matsumura K (2015). Bromoenol lactone, an inhibitor of calcium-independent phospholipase A2, suppresses carrageenan-induced prostaglandin production and hyperalgesia in rat hind paw. Mediat Inflamm.

[25] Bhattacharjee A, Majumder S, Das S, Ghosh S, Biswas S, Majumdar S (2016). *Leishmania donovani*-induced prostaglandin E2 generation is critically dependent on host toll-like receptor 2-cytosolic phospholipase A2 signaling. Infect Immun.

[26] França-Costa J, Van Weyenbergh J, Boaventura VS, Luz NF, Malta-Santos H, Oliveira MCS (2015). Arginase I, polyamine, and prostaglandin E_2_ pathways suppress the inflammatory response and contribute to diffuse cutaneous leishmaniasis. J Infect Dis.

[27] Saha A, Biswas A, Srivastav S, Mukherjee M, Das PK, Ukil A (2014). Prostaglandin E_2_ negatively regulates the production of inflammatory cytokines/chemokines and IL-17 in visceral leishmaniasis. J Immunol.

[28] Connelly MC, Kierszenbaum F (1984). Modulation of macrophage interaction with *Trypanosoma cruzi* by phospholipase A_2_-sensitive components of the parasite membrane. Biochem Biophys Res Commun.

[29] Li J, Padigel UM, Scott P, Farrell JP (2002). Combined treatment with interleukin-12 and indomethacin promotes increased resistance in BALB/c mice with established *Leishmania major* infections. Infect Immun.

[30] Mendes CT, Gattaz WF, Schaeffer EL, Forlenza OV (2005). Modulation of phospholipase A2 activity in primary cultures of rat cortical neurons. J Neural Transm (Vienna).

